# The role of *MYH* and microsatellite instability in the development of sporadic colorectal cancer

**DOI:** 10.1038/sj.bjc.6603421

**Published:** 2006-10-10

**Authors:** A Colebatch, M Hitchins, R Williams, A Meagher, N J Hawkins, R L Ward

**Affiliations:** 1 Department of Medical Oncology, St Vincent's Hospital, Victoria Street, Darlinghurst, NSW 2010, Australia; 2 School of Medicine, University of NSW, Sydney 2052, Australia; 3 Department of Colorectal Surgery, St Vincent's Hospital, Victoria Street, Darlinghurst, NSW 2010, Australia; 4 School of Medical Sciences, University of NSW, Sydney 2052, Australia

**Keywords:** *MYH*, colorectal cancer, microsatellite instability

## Abstract

Biallelic germline mutations in *MYH* are associated with colorectal neoplasms, which develop through a pathway involving somatic inactivation of APC. In this study, we investigated the incidence of the common *MYH* mutations in an Australian cohort of sporadic colorectal cancers, the clinicopathological features of *MYH* cancers, and determined whether inactivation of mismatch repair and base excision repair (BER) were mutually exclusive. The *MYH* gene was sequenced from lymphocyte DNA of 872 colorectal cancer patients and 478 controls. Two compound heterozygotes were identified in the cancer population and all three cancers from these individuals displayed a prominent infiltration of intraepithelial lymphocytes. In total, 11 heterozygotes were found in the cancer group and five in the control group. One tumour from an individual with biallelic germline mutation of *MYH* also demonstrated microsatellite instability (MSI) as a result of biallelic hypermethylation of the *MLH1* promoter. Although *MYH*-associated cancers are rare in a sporadic colorectal population, this study shows that these tumours can develop through either a chromosomal or MSI pathway. Tumours arising in the setting of BER or mismatch repair deficiency may share a biological characteristic, which promotes lymphocytic infiltration.

*MYH*-associated polyposis (MAP) is an autosomal recessive condition (Al-Tassan *et al*, 2002) associated with the development of multiple adenomas and carcinomas of the colon and rectum, as well as other possible malignancies and extra-colonic manifestations (Enholm *et al*, 2003; Sampson *et al*, 2003; Sieber *et al*, 2003; Gismondi *et al*, 2004; Nielsen *et al*, 2005). The syndrome is caused by germline biallelic mutations in *MYH,* a gene that codes for a DNA glycosylase involved in base excision repair (BER) (Al-Tassan *et al*, 2002). *MYH* mutations have been shown to be ethnically specific, and most (80%) Caucasian individuals with MAP possess the Y165C or G382D mutations (Enholm *et al*, 2003; Croitoru *et al*, 2004; Fleischmann *et al*, 2004; Wang *et al*, 2004; Farrington *et al*, 2005; Peterlongo *et al*, 2005). Italian populations also carry a three base pair deletion (1395delGGA) in exon 14 (Gismondi *et al*, 2004), whereas non-Caucasian populations harbour a range of different *MYH* mutations (Jones *et al*, 2002). As MAP is a recessive condition, patients may appear to have sporadic, rather than familial, colorectal cancer. However, a number of studies have shown that biallelic mutations in *MYH* account for only a minority of sporadic colorectal cancers (Enholm *et al*, 2003; Croitoru *et al*, 2004; Fleischmann *et al*, 2004; Wang *et al*, 2004; Farrington *et al*, 2005; Peterlongo *et al*, 2005). The significance of heterozygous *MYH* mutations in terms of cancer risk remains uncertain.

*MYH-*associated cancers are thought to progress through a distinct genetic pathway which does not involve microsatellite instability (MSI) (Lipton *et al*, 2003). Supporting this hypothesis, *MYH* cancers have an increased frequency of somatic transversion mutations of APC and K-*ras* (Al-Tassan *et al*, 2002; Jones *et al*, 2002; Lipton *et al*, 2003; Jones *et al*, 2004), although no pathologically distinctive features have been described to date.

In this study, we utilised a large and well-characterised tumour bank of sporadic colorectal cancers to investigate the clinicopathological features of *MYH* cancers, and to determine whether mismatch repair and base excision repair are mutually exclusive.

## MATERIALS AND METHODS

### Patients and specimens

This study was performed with the approval of the St Vincent's Human Research Ethics Committee. After obtaining informed consent, 872 individuals undergoing complete (RO or R1) surgical resection of 893 colorectal cancers at St Vincent's Hospital, Sydney, were entered in this prospective study (Ward *et al*, 2005). The study population consisted of 402 female and 470 male subjects with a mean age of 69.2±12.1 years (range 29–99 years). Enrolment was from 1 January 1994 to 29 May 2004, and patients presenting for resection of cancer in the setting of known inflammatory bowel disease, FAP and HNPCC were not enrolled. Peripheral blood was also collected from 284 male and 194 female healthy blood donors (Red Cross Blood Bank, Sydney), with a mean age of 45±14 years.

Tumour stage was assessed according to AJCC/UICC guidelines and histopathological characteristics were determined as previously described (Ward *et al*, 2005). Microsatellite status was determined using primer sets for Bat 25, Bat 26, Bat 40, D5S346, D2S123 and D17S250, and tumours with instability at two or more markers were classified as microsatellite unstable, the remainder being categorised as microsatellite stable (Ward *et al*, 2005). Immunohistochemistry for the mismatch repair proteins and p53 was performed as previously described (Ward *et al*, 2005).

### *MYH* mutation analysis

To identify the common ‘Caucasian’ pathogenic *MYH* mutations, exons 7, 13 and 14 of *MYH* were amplified and sequenced from lymphocyte DNA using previously designed primers (Gismondi *et al*, 2004; Kairupan *et al*, 2005). The Y165C (exon 7) and 1395delGGA (exon 14) mutations were confirmed by sequencing the complementary strand, and the G382D (exon 13) mutation was verified using a restriction enzyme digest (*Bgl*II). The remaining *MYH* exons were then sequenced in those individuals who were heterozygotes for one of the common mutations. Briefly, exon-specific primers ((Gismondi *et al*, 2004; Kairupan *et al*, 2005) additional details available on request) were used to amplify the 16 exons of *MYH* in 25 *μ*l reactions containing 100 ng of genomic DNA, 0.4 *μ*M of each of the primer, 0.2 *μ*M of dNTP, 1 U of FastStart *Taq* polymerase and a PCR master mix at 1.5 mM MgCl_2_. The PCR products were purified using the QIAquick PCR purification kit (Qiagen, Basel, Switzerland) and sequencing was performed using the Big Dye Terminator Cycle Sequencing kit (Applied Biosystems, Rotkreuz, Switzerland). Purified sequence reactions were separated on the ABI3100 Genetic Analyser (Applied Biosystems), and the nucleotide sequences analysed with the aid of the Sequencher software program (Gene Codes Corporation, Ann Arbor, MI, USA).

### Methylation analysis of MLH1 promoter

DNA (1–2 *μ*g) was converted with sodium bisulphite and the ‘A’ and ‘C’ regions of the *MLH1* promoter were subject to combined bisulphite restriction analyses (COBRA) as previously described (Hitchins *et al*, 2005). Samples giving a positive restriction digestion pattern indicative of the presence of methylation were cloned into the pGEMTeasy vector (Promega, Annandale, NSW, Australia), and individual clones were fluorescently sequenced on an ABI 3100 using SP6 and T7 vector primers.

### Statistical analysis

Categorical variables were compared using the *χ*^2^-test or Fisher's exact test as appropriate. In patients with more than one cancer, only the properties of the most advanced tumour were considered in the case control study. All tumours were considered when analysing tumour properties. A probability value of less than 0.05 was considered significant. All data were analysed using the SPSS statistical software V13.0 (SPSS Inc., Chicago, IL, USA).

## RESULTS

### Frequency and spectrum of *MYH* mutations

Of the 872 individuals with colorectal cancer, two were compound heterozygotes (patient 9033 and 13714) and a further 11 had a monoallelic mutation in *MYH* (Table 1). None of the 478 control individuals harboured biallelic *MYH* mutations, but five were found to be heterozygotes (Table 1). After sequencing the remaining exons in the 16 heterozygotes, a number of nonpathogenic missense mutations were identified (one V22M, five Q324H and one S501F). No significant difference in the frequency of biallelic (*P*=0.542, Fisher's exact test) or monoallelic *MYH* mutations (*χ*^2^=0.12, *P*=0.72) was found between the colorectal and the control groups.

### Pathological features of tumours in *MYH* mutation carriers

At the time of surgical resection, one compound heterozygote (patient 9033) was found to have synchronous stage III colorectal cancers as well as more than 100 adenomatous polyps scattered throughout the colon. The clinicopathological characteristics of these cancers, as well as the stage III caecal cancer from individual 13714 (G382D/1395delGGA) are shown in Table 2. Both of these individuals harboured multiple adenomatous polyps; however the only manifestation of extra-colonic disease was in patient 9033, who had a history of malignant melanoma at the age of 28 years. Interestingly, the three cancers from these individuals displayed a prominent infiltration of intraepithelial lymphocytes.

In most respects, cancers arising in monoallelic *MYH* carriers were indistinguishable from cancers arising in individuals in whom no *MYH* mutations had been identified (Table 2). A statistically significant increase in the number of intraepithelial lymphocytes was noted in the heterozygote mutation group compared with the rest of the colorectal cohort (five of 11 *vs* 151 of 827 assessable cases, *P*=0.04, Fisher's Exact test). However, this association was probably a reflection of the microsatellite status of the cancer, as all but one case with prominent intraepithelial lymphocytes was associated with a microsatellite unstable cancer.

### Microsatellite instability and *MYH* mutations

**Figure 1:**
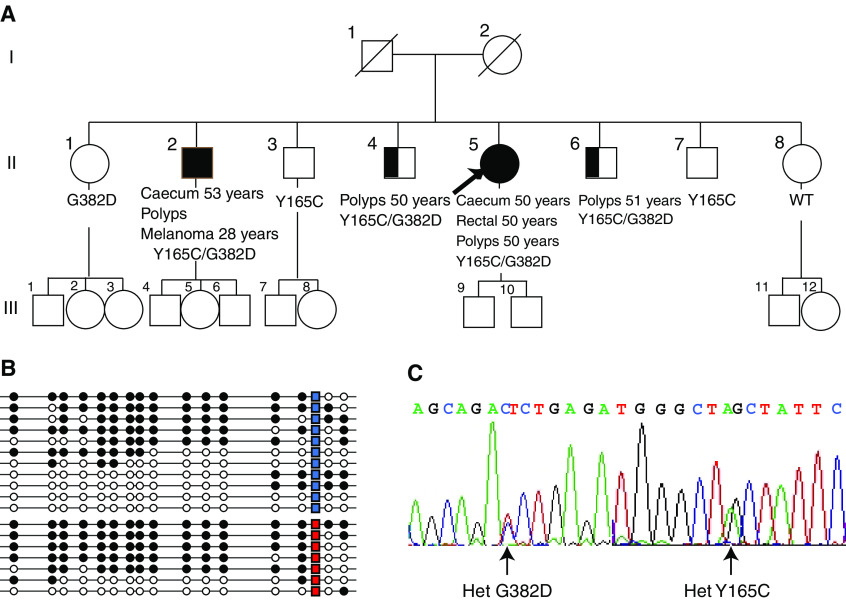
(**A**) Pedigree showing the family of the proband (case 9033, marked with black arrow) and their respective disease and mutation status. WT, wild type; left half shaded black, polyps; right half shaded black, cancer. (**B**) Schematic of promoter region of *MLH1* from the caecal tumour from case 9033 demonstrating biallelic methylation. Each allele is distinguished by the presence of an SNP (coloured square). Circles represent CpG islands, filled circles represent methylation, the blue square represents guanine and the red square represents adenine. (**C**) Sequence traces obtained from the caecal tumour of case 9033 demonstrate the presence of biallelic *MYH* mutations. The patient was compound heterozygote for the Y165C and G382D mutations.

The frequency of tumours with microsatellite instability arising in cases with MYH mutations is shown in Table 2. Of the 893 tumours assessed for MSI in this study, 139 (15.5%) showed MSI. Individuals who were heterozygous for an MYH mutation were more likely to have an MSI tumour (five of 11, *χ*^2^ test *P*<0.01), whereas one of the cancers seen in the two individuals with biallelic MYH mutations was also MSI (Table 2). This individual (9033) had a microsatellite unstable caecal cancer which failed to express the mismatch repair protein MLH1 by immunohistochemistry, as well as a rectal cancer that was microsatellite stable and expressed the mismatch repair proteins *MLH1*, *MSH2* and *MSH6*. The microsatellite unstable caecal cancer did not display the typical pathological features of a sporadic MSI colorectal cancer, in that it was nonmucinous, of low histological grade and BRAF mutation negative (data not shown). The pedigree and results of *MYH* mutation testing in this family is shown in Figure 1, panel A. Germline testing of the mismatch repair genes failed to identify a pathogenic mutation in individual 9033.

To determine the mechanism of inactivation of *MLH1* in the caecal cancer of patient 9033, we subjected the *MLH1* promoter to bisulphite sequencing. As patient 9033 was heterozygous for the A/G polymorphism (rs11800734) in the *MLH1* promoter, bisulphite sequencing was able to confirm methylation of both alleles in the tumour cells (Figure 1B). Unmethylated A and G alleles were presumably derived from contaminating normal tissue, thus suggesting that normal somatic colonic cells were unmethylated. Microdissected tumour tissue was then subjected to genetic analysis for *MYH* mutations. As expected, the tumour displayed the missense mutations Y165C and G382D (Figure 1C), thus confirming that this carcinoma possessed MSI in the context of biallelic *MYH* mutations.

## DISCUSSION

This study shows that biallelic *MYH* mutations are rare (0.2%, 2/872) in an Australian colorectal cancer population. It must be acknowledged that the screening strategy used in this study examined exons 7, 13 and 14 of MYH. While these regions account for around 80% of known mutations in Caucasians, a small number of pathogenic mutations will have been missed because of this approach. Nevertheless, this mutation frequency was comparable to studies from Finland (0.4%, four out of 1042) (Enholm *et al*, 2003), North America (0.4%, two out of 555 and two out of 444, 0.5%) (Wang *et al*, 2004; Peterlongo *et al*, 2005), Canada (1.0%, 12 out of 1238) (Croitoru *et al*, 2004), Scotland (0.5%, 12/2239)(Farrington *et al*, 2005) and Britain (0.3%, one out of 358) (Fleischmann *et al*, 2004). Like other studies, we found a lower frequency of the Y165C allele (0.2% in cases and 0.1% in controls) than the G382D allele (0.6% in cases and 0.4% in controls). Given the significant number of southern European migrants resident in Australia, it is surprising that only one individual was identified with an exon 14 mutation (135delGGA) (Gismondi *et al*, 2004). While others have suggested that the frequency mutations in exon 14 such as A459T justify routine mutation screening of this exon, our data does not support this position, at least in a nonpolyposis population (Alhopuro *et al*, 2005).

Lipton *et al* (2003) proposed that carcinomas arising in the setting of biallelic *MYH* mutations followed a distinct genetic pathway. If this hypothesis was correct, a distinctive clinicopathological phenotype may accompany the development of *MYH*-related cancers. Although we found little evidence to support this suggestion, it was interesting that the three cancers arising in the individuals with biallelic mutations in *MYH* displayed a prominent infiltration of intraepithelial lymphocytes. Lymphocytic infiltrates are strongly associated with the development of mismatch repair-deficient cancers, although a proportion of microsatellite stable tumours also demonstrate intraepithelial lymphocytes (Ward *et al*, 2001). The mechanism for recruitment of lymphocytes in these cancers is unknown (Quinn *et al*, 2003), but mutator and DNA repair phenotypes may share properties which favour the retention of lymphocytes in the epithelium. The phenomenon of increased lymphocytic infiltration in MSI tumours has been suggested as a possible reason for the improved survival seen in these cancers (Guidoboni *et al*, 2001). It is possible that MAP cancers represent a subgroup of MSS cancers that may also share this improved prognosis.

Significantly, our study has also shown that MSI cancers can arise in the setting of biallelic germline *MYH* mutations, and that MSI cancers may arise more commonly in individuals with monoallelic MYH germline mutation than in normal individuals. To date, the 168 MAP tumours (includes 48 colorectal cancers) reported in the literature were microsatellite stable (Lipton *et al*, 2003; Fleischmann *et al*, 2004; Wang *et al*, 2004; Nielsen *et al*, 2005). This is expected given that mutations of *MYH* predisposes to inactivation of APC through G:C to A:T transversions (Al-Tassan *et al*, 2002). The absence of MSI in MAP carcinomas led to the suggestion that the derangement of both base excision repair and mismatch repair was not compatible with cellular survival (Kambara *et al*, 2004). The case of an MSI cancer in the setting of MYH biallelic germline mutation reported in our study clearly does not support this hypothesis, and it is interesting that *MLH1* was inactivated by biallelic promoter methylation, rather than through somatic mutations secondary to loss of MYH function. In this case, it is apparent that biallelic *MYH* mutations have played a role in the early stages of colorectal carcinogenesis through APC mutation and polyp formation. Yet, this influence has been superseded by the tumorigenic drive provided by loss of *MLH1* function. It is possible that MYH mutations may have a largely permissive role in tumorigenesis through polyp formation, with subsequent progression occurring as a result of changes to other key genes. This role of MYH mutations in colorectal carcinogenesis is consistent with the finding in murine models that MYH knockouts are phenotypically normal (Xie *et al*, 2004).

It is also worth noting that patient 9033 was only 50 years of age. This is a strikingly young age for the development of a ‘sporadic’ MSI cancer through biallelic methylation of *MLH1* (Ward *et al*, 2001), and raises the possibility that *MYH* mutation has somehow either facilitated methylation, or acted synergistically with MLH1 loss to more rapidly drive tumour progression. A more testable implication is that *MYH* mutation may be worth considering in younger individuals with MSI tumours that have developed because of *MLH1* methylation. Such individuals are increasingly likely to be recognised because of immunohistochemical testing of colorectal cancers. Biallelic *MYH* mutation remains an uncommon event, and the small numbers of cases in this report allow us only to speculate on the biological relevance in a sporadic population. More systematic studies of *MYH* status in distinct populations will be required to properly evaluate the true significance of *MYH* loss in colorectal carcinogenesis.

## Figures and Tables

**Table 1:** List of *MYH* mutations and polymorphisms in the colorectal cohort and the control group

**ID**	**Age**	**Gender**	***MYH* mutations**		**Polymorphism**
*Colorectal patients*					
Biallelic (*n*=2)					
9033	50	F	Y165C	G382D	NT
13714	60	M	G382D	1395delGGA	NT
					
Monoallelic (*n*=11)					
15311	77	F	Y165C		
11408	88	F	G382D		
10355	76	M	Y165C		
13122	72	M	G382D		Q324H
11358	44	M	G382D		Q324H
13911	81	F	G382D		
14359	70	M	G382D		
11913	88	F	G382D		
14838	64	F	Y165C		Q324H, S501F
511	74	F	G382D		
11328	65	F	G382D		
					
*Controls*					
Monoallelic (*n*=5)					
	69	M	G382D		Q324H
	54	M	G382D		Q324H
	62	F	Y165C		V22M
	31	M	G382D		
	58	M	G382D		

NT=not tested; M=male; F=female.

**Table 2:** Clinicopathological features of individuals with germline *MYH* mutations

**Patient ID**	**Adenomas**	**Cancer site**	**Stage**	**Grade**	**Mucin**	**IELs**	**P53 IHC**	**MSI**
*Biallelic mutation carriers*								
13714	Seven TA, One HP	Caecum	III	Low	No	Yes	Neg	MSS
9033	Caecal carpet	Caecum	III	Low	No	Yes	Neg	MSI
	>100 adenomas	Rectum	III	Low	Yes	Yes	Pos	MSS
								
*Monoallelic mutation carriers*								
15311	No	Caecal	II	Low	No	No		MSI
11408	NS	AC	II	Low	No	Yes	Neg	MSS
10355	No	DC	II	High	Yes	Yes	Neg	MSI
13122	No	Caecum	I	High	No	Yes		MSI
11358	No	Rectum	III	Low	No	No	Neg	MSS
13911	No	Caecum	II	Low	No	No	Neg	MSS
14359	No	TC	II	Low	Yes	Yes		MSI
11913	Yes	Caecum	II	Low	No	Yes	Neg	MSI
14838	No	AC	IV	Low	No	No		MSS
511	NS	Rectum	III	Low	No	No	Pos	MSS
11328	No	SC	II	Low	No	No	Neg	MSS

MSS=microsatellite stable; MSI=microsatellite instability; IELs=prominent intraepithelial lymphocytes (>6 per high-power field); TA=tubular adenoma; HP=hyperplastic polyp; AC=ascending colon; TC=transverse colon; DC=descending colon; SC=sigmoid colon.
